# Europeanisation, Sovereignty and Contested States: The EU in northern Cyprus and Palestine

**DOI:** 10.1177/1369148117727534

**Published:** 2017-09-28

**Authors:** Dimitris Bouris, George Kyris

**Affiliations:** 1 Department of Political Science, University of Amsterdam, The Netherlands; 2 Department of Political Science and International Studies, University of Birmingham, Edgbaston, UK

**Keywords:** conflict, contested states, European Union, Europeanisation, sovereignty, statehood

## Abstract

Combining the literature on sovereignty and Europeanisation, this article investigates the engagement and impact of the European Union (EU) on contested states (states lacking recognition) through a comparative study of the Turkish Republic of Northern Cyprus (TRNC) and Palestine. We find that characteristics of contested statehood mediate EU engagement and impact: the lack of international recognition limits EU’s engagement but encourages development promotion, international integration and assistance of local civil society. Lack of territorial control limits engagement, but ineffective government offers opportunities for development promotion and state-building. As such, and in addition to offering a rich empirical account of two prominent contested states, the article contributes to the discussion of international engagement by developing an innovative conceptual framework for understanding EU’s impact on contested states—a topic neglected within a literature dominated by conventional statehood or conflict resolution themes but very important given extensive international engagement in contested states—and related conflicts.

## Introduction

The post-Maastricht re-launch of European Union’s (EU) international relations, the 2004–2007 enlargement and the subsequent redrawing of its external borders brought the EU closer to a range of conflicts and a rather awkward type of states: self-declared states, which are not recognised by a significant part of the international community. Indeed, the majority of conflicts in the EU’s near abroad relate to such contested states, like Kosovo, Palestine, the Turkish Republic of Northern Cyprus (TRNC), South Ossetia and Abkhazia, Transnistria and Nagorno-Karabakh in the post-Soviet space or, more recently, separatism in Donetsk and Luhansk in Ukraine. Further away, the Sahrawi Arab Democratic Republic (SADR) in Western Sahara, Somaliland and Taiwan are also invariably important for the international role of the EU, while the possibility of an independent but unrecognised Kurdistan shows the ongoing significance of contested statehood in international politics.

Although the literature has tried to conceptualise the EU’s conflict resolution role (see Diez et al., 2008; Tocci, 2007; Whitman and Wolff, 2012), the issue of contested statehood and its implications for EU engagement remain under-researched. While some works on contested states have touched upon the so-called ‘engagement without recognition’ (see for example Caspersen, 2015; Cooley and Mitchell, 2010; Ker-Lindsay, 2015), EU studies have mainly focused on the impact of integration on the state from which the contested state attempts secession (Coppieters et al., 2004; Diez et al., 2008), how domestic actors of contested states understand the EU (Popescu, 2007; Vahl and Emerson, 2004), diplomatic issues (Papadimitriou and Petrov, 2012) or the EU’s efforts for state-building but without taking into account contested statehood (Bieber, 2011; Börzel, 2011; Bouris, 2014). In this regard, this article addresses this gap in the literature by offering a comparison of the TRNC and Palestine in order to answer the following central research question: How do different parameters of contested statehood mediate the impact of the EU on contested states?

The article focuses on contested states as those entities that have declared independence, but are not recognised by a significant part of the international community, and which also display at least some degree of what are conventionally understood as statehood characteristics: a certain population, a territory, a government and capacity to enter into relations with third states (see Geldenhuys, 2009). Conceptually, the article draws upon debates on Europeanisation and sovereignty, the combination of which helps to account for how the impact of the EU is mediated by a set of parameters in contested states, namely, lack of international recognition, effective government and territorial control. Policy documents, official statements and a series of semi-structured interviews with EU officials and local elites involved with EU policies in Brussels, Nicosia, Jerusalem and Ramallah are analysed qualitatively. These interviews, dating back to crucial eras of EU involvement, help to triangulate the rest of material collected and provide new empirical insights. They also support the key argument of this study: that the role and impact of the EU are compromised either because the lack of international recognition does not allow the development of meaningful relations and because the lack of territorial control obstructs/limits the EU’s ability to apply its policies on the ground. Yet we also find certain opportunities for the EU, namely that ineffective government allows the promotion of state-building, while non-recognition encourages the empowerment of civil society and/or greater international integration.

As such, the contribution of this article is twofold. First, it introduces an innovative framework that draws upon both international relations and European studies to offer a systematic conceptualisation of the Europeanisation of contested states, which, although highly important and topical, remains relatively under-researched. While not exploring in depth the relationship between Europeanisation process and the conflict trajectory, our analysis raises a range of questions on this relationship and aims to offer a blueprint for further research on the links between contested statehood, international engagement and conflict resolution. Second, the article offers a rich empirical account of the EU’s role in two prominent contested states. The article is structured as follows. The next section reflects on the conceptual framework and research design, and it is followed by a section that focuses on the two case studies and, finally, a section offering comparative insights and avenues for further research.

## Europeanisation and the different faces of sovereignty

In exploring the EU’s involvement and impact on contested states, this article engages with the Europeanisation literature, which has focused on EU-induced changes in national policy, institutions and politics (Börzel, 1999; Ladrech, 1994; Töller, 2010; Wallace, 2000). Having started as a debate regarding the impact of the EU on member states, Europeanisation scholars have also discussed the EU’s role vis-à-vis candidates for accession (Glenn, 2004; Grabbe, 2001; Sedelmeier, 2011) or third states (Lavenex and Uçarer, 2004: 419; Schimmelfennig, 2009: 8; Wallace, 2000: 371). Europeanisation is seen as a process of structural change that affects actors, institutions, interests, practices and ideas (Featherstone and Radaelli, 2003) and comes as a response to EU policies and decisions. In terms of how this process of Europeanisation takes place, the literature points to three mechanisms, which guide the conceptual analysis of our empirical findings. First, Europeanisation can be a result of compliance with institutional or policy directions. Earlier Europeanisation works focused on institutional compliance as a result of EU law implementation for existing (see for example Knill and Lehmkuhl, 2002) or candidate EU member states (Schimmelfennig and Sedelmeier, 2004). However, pressures for institutional compliance can also be exerted upon third states not as a result of legal pressures to implement the acquis but because this is the only way for these countries to further their relations with the EU—this becomes relevant to this study, which focuses on cases outside the EU. In this context, studies have also looked at how neighbours of the EU might comply with certain EU standards (Lavenex, 2008; Schimmelfennig, 2009). Second, drawing on rational choice institutionalism, Europeanisation studies have also focused on changes to domestic opportunity structures, that is the distribution of power (Börzel and Risse, 2000). Finally, sociological institutionalism reflects on the socialisation of actors into certain practices (Börzel and Risse, 2000; Schmidt, 2001: 6) and how the EU can change domestic beliefs and expectations, styles, practices and ‘ways of doing things’ (Radaelli, 2000: 44).

Because of the focus on contested *states*, this article considers state institutions as the starting point of analysis, although we find broader implications for the areas of civil society and political elites that are important to note vis-à-vis institutional changes. In this regard, we adopt a two-level analysis, discussing the engagement of the EU in contested states but focusing mainly on the impact of this engagement, which is central to the discussion of Europeanisation. A recurring theme within this debate has been how Europeanisation is mediated by national parameters, including a ‘misfit’ between what exists at the domestic level and the reforms promoted by the EU (Börzel and Risse, 2000; Cowles et al., 2001; Featherstone and Radaelli, 2003). It is this focus on how different national parameters mediate the impact of the EU that also makes Europeanisation a useful tool in understanding the EU’s engagement in contested states and the implications that their unique characteristics have for this engagement. This has been explored before (see Kyris, 2013, 2015), but the contribution of this article is that we further this Europeanisation discussion by combining it more explicitly and systematically with the concept of sovereignty, which helps identify lack of international recognition, territorial control and effective government as certain parameters of contested statehood and explores how they might mediate EU impact. We turn to the discussion on sovereignty because of how central it is in the way states, contested or not, are approached by both scholars and international actors, like the EU.

While the ways in which it informs practice in world politics have changed over the years (see Jackson, 2007), the idea of sovereignty remains relatively stable and central to how we understand statehood. In a seminal conceptualisation of statehood, Krasner (2001) distinguishes between external (also referred to as negative—see Jackson, 1993) sovereignty and internal (or positive) sovereignty. While external sovereignty refers to the recognition of a state from outsiders in the international system, internal sovereignty refers to effective state structures and authority—what is often described as empirical statehood.

It follows from this that what is seen as the sine qua non characteristic of contested statehood is the lack of external sovereignty—that is, the fact that these entities are not recognised as states by a significant part of the international community. Often resulting in extensive international isolation, the lack of statehood recognition should not be confused with the recognition of the *right* to statehood—what Geldenhuys (2009) calls ‘titular’ recognition. Many works on contested states have engaged with the concept of sovereignty (see Caspersen, 2012; Caspersen and Stansfield, 2011), and it is important to underline here that there exist many forms of interaction, such as trade, air and postal communication (Berg and Toomla, 2009) or membership of international organisations (Ker-Lindsay, 2012, 2015), which, although not constituting external sovereignty per se, might add to sovereignty claims on behalf of contested states. Applying those ideas to understand contested states, we suggest that there is high external sovereignty where there is recognition by more than two-thirds of United Nations (UN) member states and low external sovereignty where less than one-third of UN members recognise, with the rest of contested states enjoying medium external sovereignty. This approach should be combined with a more qualitative analysis, which also accounts for titular recognition. As a result, we consider TRNC as having low external sovereignty because it is only recognised by Turkey, while Palestine is considered to have high external sovereignty because it is recognised by more than two-thirds of UN members and it also enjoys widespread titular recognition.

Contested states should also be conceptualised as demonstrating some lack of internal sovereignty—that is, de facto effective control of the government of the state over its territory and people and, generally, effective economic and political systems and institutions (Clapham, 1998; Krasner, 2004). While this issue has been extensively discussed with reference to ‘weak’, ‘quasi’ or ‘failed’ states (Jackson, 1993; Migdal, 1988), we argue that it gains increased importance in cases of contested states. Often, territorial disputes and/or secession efforts come with lack of control of the contested state government over its self-proclaimed territories. This is because the parent or reference state^1^ might be able to exercise control over those areas, such as in the case of Palestine. In 1993, the Oslo Accords created the Palestinian Authority (PA), tasked to control a number of non-contiguous population centres. With Oslo II, the West Bank was divided into three areas: it was only in Area A (17.7%) that the PA was given full administrative and security control. In Area B (21.3%), the PA was given civil control while Israel maintained security control. In Area C (61%), Israel would retain full responsibility and control.

But internal sovereignty also relates to the presence of effective governments, and the relatively young character of many of the existing contested states (8 of 12 declared independence after 1983), coupled with the lack of international integration, might also result in weak state apparatuses. Palestine, for example, can be considered as having compromised internal sovereignty also in this respect. Linked to the fact that the state formation process only started in 1993 (before this the West Bank and Gaza Strip were under full Israeli Civil Administration Control), Palestine should be considered as having low internal sovereignty by the time the EU embarked on engagement. TRNC, on the other hand, can be considered as displaying high internal sovereignty. This is because TRNC is a stable political and economic system, including a centralised and effective government, public administration, a multi-party political system and a working economy and also effective control of the territories they claim (northern Cyprus). Turkish Cypriot dependency on Turkey for performing certain state functions (e.g. reliance on Turkish military for security) is crucial, but this dependency does not undermine their overall internal sovereignty, and as such, it is not at the focus of our discussion here. Interestingly, these two faces of sovereignty seem to be interrelated. For example, Caspersen (2015) has looked at strategies of ‘earned sovereignty’ and how contested states might seek to adopt values promoted by the international community with the hope to increase their external sovereignty, a strategy which can eventually reinforce what is understood as internal sovereignty.^2^

In this regard, a series of questions are raised as to whether processes of Europeanisation are mediated by different degrees of external and internal sovereignty. The existing literature on international engagement highlights certain problems that mostly relate to external sovereignty issues, particularly the fact that engagement is difficult because it is seen as recognition by implication, a problem that becomes especially acute where there is a parent state whose territorial integrity is prioritised and which is keen to veto engagement (see, for example, Herrberg, 2010; Ker-Lindsay, 2012). The literature has, therefore, found a ‘reluctant’ engagement (Caspersen, 2008)—but engagement nevertheless—which among others focuses on involving local leadership and civil society, especially moderates, and in peace processes (Berg and Pegg, 2016). In this regard, our analysis aims to discuss the different obstacles and also opportunities, thus also adding directly to the literature on ‘engagement’. Our study of the TRNC, which is heavily unrecognised, will seek to add to those analyses by moving away from discussion of broader engagement from the side of the international community and exploring EU engagement in specific and in more depth. What is more, by combining insights from the discussion on contested states and Europeanisation, this article also contributes to the literature by exploring not only the EU’s engagement per se but also its impact on the ground and how it is mediated by parameters that relate more to internal sovereignty, that is, state structures and territorial control. The focus on those internal characteristics of unrecognised states, tested in our case study of Palestine, is yet another way in which this article adds to the literature, which has been dominated by more external issues of international recognition.

To this end, our analysis draws on the conceptual discussion of sovereignty to account for how the lack of (a) international recognition and (b) territorial control and effective government (independent variables) mediate the engagement and impact of the EU (dependent variable) on TRNC and Palestine, respectively, which is posited to unravel via policy or institutional compliance, redistribution of power and/or changes in ideas and practices (casual mechanisms) offered by the Europeanisation literature. We look at the EU impact that has been shaped by parameters of contested statehood rather than EU-induced changes more generally. To ensure causality, we also focus exclusively on EU policies (rather than other processes, such as engagement from different international organisations; see also Moumoutzis and Zartaloudis, 2016). We explore our research question and test our independent variables (a) and (b) in the two cases of the TRNC and Palestine separately and then we compare our findings. This is done because although Palestine and TRNC are both contested states, they differ significantly when we look closer at the characteristics of contested statehood with regard to the independent variable of this study: while TRNC lacks predominantly international recognition (independent variable a), Palestine has a greater deficit of territorial control and effective government (independent variable b). As a result, this variation with high and low values of the independent variable (high external sovereignty and low internal sovereignty in the TRNC, vice versa in Palestine) is especially conductive to heuristic case studies like these ones and allows us to uncover differences in the dependent variable and the casual mechanisms (George and Bennet, 2005). What is more, the choice of these two cases is also important for illustrating two more, related points often found in the literature: first, that sovereignty can have different degrees and should not be seen as absolute (see also Caplan, 2006: 12); second, that different degrees of internal and external sovereignty might co-exist. The small number of cases will allow to explore in depth the process of Europeanisation rather than simply its occurrence (Moumoutzis and Zartaloudis, 2016: 344–346; see also Van Era, 1997: 55 on cases studies). By doing so, we contend that our findings are an important starting point for exploring process of Europeanisation in the variety of contested states that exist today.

## The EU in the TRNC

The failure of the bi-communal Republic of Cyprus (RoC) established in 1960 led to conflict and the gradual territorial and administrative division of the island into two zones: the RoC is now monopolised by the Greek Cypriots in the south of the island, while in 1983 Turkish Cypriots officially self-declared their secessionist TRNC. Since then, the TRNC has had effective control of northern Cyprus, but is only recognised by Turkey. Conversely, RoC continues to be recognised as the only legitimate government of the country. So far, efforts to resolve the Cyprus conflict based on a federal reunification have failed. Following accession of Cyprus, the engagement of the EU with the Turkish Cypriots has had both practical and political reasons: officially, the whole of the island is an EU territory, but, in practice, northern Cyprus remains an area that the government of the RoC cannot control. For that reason, EU law is suspended in the north (Protocol 10 of the Accession Treaty). Yet the persisting potential for the resolution of the Cyprus conflict based on reunification means that the EU needs to prepare northern Cyprus for its full European integration, when a federation would replace the RoC as a member state. In this regard, the EU has tried to increase the chances of a successful resolution mostly by means of the Financial Aid Regulation (FAR) (Council of the European Union, 2006), which aims to assist development and preparation for EU law implementation, and the Green Line Regulation (GLR) (Council of the European Union, 2005b), which facilitates trade between the two communities.

From the beginning, contested statehood and the low degree of external sovereignty put hermetic barriers between EU engagement and impact in northern Cyprus. The Commission’s plan for a preferential trade agreement between the EU and northern Cyprus was never implemented, largely because the Greek Cypriot–led RoC asserted that implementation would imply recognition of the TRNC. But lack of recognition has impacted even those instruments that have been adopted for northern Cyprus^3^ and has shaped the effect of the EU on the ground, often undermining the success of EU initiatives (TAIEX, 2009: 18). For example, because TRNC is not recognised, the Commission cannot use financial agreements with the local government as the legal basis for the aid provided. Instead, the EU had to respond with a more direct engagement, which tested its resources (European Court of Auditors, 2012: 2).

In an effort to overcome the conundrum of dealing with an unrecognised state, some novel institutional solutions were introduced. While the government of the contested state enjoys an important role in the interaction with the EU and was required to make adjustments in this regard (in a similar fashion to third states or candidates for accession—for more on institutional changes, see Kyris, 2015), the low degree of external sovereignty is responsible for the extensive role of local technocrats and civil society in managing EU affairs. For example, the highly technocratic EU Coordination Centre was established by the Turkish Cypriots in order to save Brussels from the risk of ‘recognition by implication’.^4^ Similarly, the EU has also opted for dealings with civil society. An interesting example here is the role that the Turkish Cypriot Chamber of Commerce (KTTO) has gained in the GLR. The implementation of the regulation called for a series of trade tasks, such as the issue of trade documents and monitoring of trade, usually given to national ministries.^5^ In this instance, however, the EU preferred to avoid formal interactions with public institutions and delegated these responsibilities to the KTTO (European Commission, 2004), which in this way gained an unpredictably important influence over certain policies. Because of this, the KTTO established a strong lobbying office in Brussels, operating beyond the scope of the GLR strictly speaking. The KTTO is only one example of how the lack of external sovereignty has mediated the process of Europeanisation in the form of changing domestic power distribution (Europeanisation mechanism 2) towards an empowered civil society as an alternative interlocutor with Brussels. Indeed, members of the European Parliament Group for the Turkish Cypriots also discuss how they tend to engage more with civil society, as a result of the non-recognition of the self-declared state.^6^ To this end and while the process of European integration has tended to favour national executives, in the contested TRNC the executive has an important role in dealing with the EU—but so do non-state actors, like civil society or technocrats, who therefore enjoy policy knowledge and influence and better access to the international and EU environment.

Besides, the overall objective of the FAR is to facilitate the reunification of Cyprus by encouraging Turkish Cypriot development, alignment with EU law and more contacts with the Greek Cypriots (Council of the European Union, 2006: 1.1). Towards these aims, the EU has provided financial assistance and expert seminars on issues such as preparation for legal approximation with EU law or training on the principles of effective transposition of legal texts (TAIEX, 2009: 18) and, generally, has facilitated the exchange of best practices and techniques (TAIEX, 2004: 12, 2005: 10). Here, the low degree of external sovereignty seems conducive to EU-induced reform of policy and administrative structures: because of the limited recognition, there is an important international isolation that comes with often outdated^7^ institutions and the misfit between Turkish Cypriot policies, practices and procedures and what EU integration calls for.^8^ This situation leaves space for significant Europeanisation pressures to be exerted. However, because it is unclear if and when the island will reunify (which would make EU law applicable also in northern Cyprus), there are no strong institutional compliance pressures (mechanism number 1), neither does the EU prescribe very explicit institutional models. Instead, changes that occur can be better understood as an outcome of the socialisation of Turkish Cypriot elites with EU actors, such as in the context of TAIEX, whose activities have anyway focused on the transformation of the local mentality with regard to its adjustment to EU policies and practices.^9^ Positive assessment from the side of the EU with regard to progress in a number of areas (such as environment, financial services—see TAIEX, 2007: 10, 2008: 20), as well as the approval of a range of new laws (for example competition law, health and safety) as a result of the FAR, suggests a process of Europeanisation through changes to practices and ‘ways of doing things’ (mechanism number 3), yet a far greater potential for change seems to be unrealised. This is because the EU cannot work as easily with authorities of the contested state (Court of Auditors, 2012: 12) as well as because of the unclear prospects of reunification and application of EU law in northern Cyprus.

Similarly, the low degree of external sovereignty and the underdevelopment that has been caused by the non-recognition and isolation of the Turkish Cypriots have also shaped the way the EU has tried to assist socio-economic development. In an effort to boost infrastructure, the EU has funded a projects on waste management, traffic safety and energy matters (European Court of Auditors, 2012: 5). The EU has also targeted development through grant schemes like Improving Agricultural Production. Other programmes have aimed at technical assistance and capacity-building, also through the inclusion of locals in EU-level processes. At the same time, the Commission also underlines problems that stem from international isolation and underdevelopment, such as an extraordinary demand for supervision or the beneficiaries’ lack of experience of claiming and managing EU grants (European Court of Auditors, 2012: 5). These problems can be considered as compromising the overall impact that the EU could achieve by fulfilling the objectives of the regulation.

Within development assistance, capacity-building of local civil society has been identified as a specific priority of the EU (Civil Society Support Team, 2009). Through the Cypriot Civil Society in Action programmes, the EU has sought to help locals, also in the context of reconciliation with the Greek Cypriots. Technical help seems to have been particularly important for the Turkish Cypriots. For example, because projects of other donors (e.g. UN) have traditionally been supervised by externals, the EU made an extra effort to increase the capacity of the non-governmental organisations (NGOs) to maintain ownership of their activities.^10^ These efforts are important for the Europeanisation via change of practices and ideas (mechanism number 3) of those actors involved towards strengthening their internal organisation capacities and staff skills (Business and Strategies European Consortium, 2013: 34). By doing so, the EU’s assistance has also empowered local civil society (see Europeanisation mechanism number 2) and, in some instances, has allowed them to exit the constraints of the domestic arena (Goetz and Hix, 2001: 12) to pursue their objectives. For example, the EU has funded the bi-communal initiative Cyprus Island–Wide NGO Development Platform (CYINDEP), which now participates in international platforms (Civicus, 2011). More locally, environmental organisations were invited to consult the government in the preparation of legislation aligned to EU law.^11^ In this sense, the EU has often opened new avenues for influencing politics. This process is particularly crucial because influencing domestic affairs has been rather challenging (Business and Strategies European Consortium, 2013; INTRAC, 2011: 34). Despite those small positive steps, there is still a long way to go, and the long-term sustainability of a powerful civil society is questionable (European Court of Auditors, 2012: 23), particularly because the Turkish Cypriot political and legal system is not conducive.^12^

Last but not least, the FAR also aims at bringing locals ‘closer to the EU’ (Council of the European Union, 2006: Article 2) via specific instruments, the rationale of which is closely related to the issue of contested statehood:

From [their] isolation resulted a remarkable deficit of knowledge about the EU … It is therefore appropriate to enable the Turkish-Cypriots … to develop fruitful relations with other EU Member States (Council of the European Union, 2006: Article 1.1).

Although the locals’ unfamiliarity with EU practices has resulted in waves of frustration with what is seen as a time-consuming process of grant applications,^13^ the promotion of youth exchanges and people-to-people contacts is a good example of how the EU assisted local organisations, who were the main beneficiaries via awarded projects such as participation in international festivals and study visits to the EU. The first call for proposals for the scheme in 2007 was rather unsuccessful, and for the second call in 2009, the Commission devoted extra resources (European Commission, 2009) in order to increase the capacity of the locals as far as preparing applications was concerned, which was considered weak and a reason for the bad results of the first round (European Commission, 2009). Indeed, more than 85% of the second call grants were successfully claimed, offering evidence as to how the EU has increased the technical capacity and professionalism of local civil society. In this regard, low external sovereignty and the resulting isolation not only facilitated an EU-informed empowered civil society (mechanism number 2) with more opportunities for links abroad, but the technical assistance towards preparing funding bids became an avenue for the communication of new ideas and practices (mechanism number 3). However, and as discussed before, the long-term sustainability of this impact in such an unstable country is questionable.

## The EU in Palestine

The reasons for the contested statehood of Palestine can be traced back to the collapse of the Ottoman Empire and the subsequent British withdrawal from these territories. In Resolution 181, the UN decided upon the division of Palestine into two states, Arab and Jewish, and the internationalisation of Jerusalem. In the aftermath of the 1967 War, Israel occupied the West Bank (including East Jerusalem), Gaza and the Golan Heights. On 15 November 1988, Yasser Arafat, President of the Palestinian Liberation Organization (PLO), proclaimed the State of Palestine based on UN Resolution 181. In 2012, the UN decided to upgrade Palestine from ‘non-member observer entity’ to ‘non-member observer state’ with a majority of 138 states voting in favour. The upgrade reconfirmed that Palestinians enjoy a certain degree of ‘titular’ recognition. Linked to the fact that 137 states already recognise Palestine, it enjoys a higher degree of external sovereignty compared to the TRNC. Conversely, it is the lack of internal sovereignty, in the form of weaker state structures and control over the territory that the Palestinians claim, which mainly defines Palestine and is explored in relation to the process of Europeanisation here.

As mentioned above, the 1993 Oslo Accords created a number of non-contiguous population centres and areas upon which the PA did not have full control. Coupled with the fact that no formal institutions existed before Oslo entrenched the low degree of internal sovereignty of Palestine, although, at the same time, this situation also provided the EU with state-building opportunities. The EU provided half the funding needed for the setting up of the PA’s institutions following the 1993 Oslo Accords because it was hoped that building Palestinian institutions would be a first step towards the establishment of a Palestinian state and the end of the conflict (Bouris, 2014: 73; Le More, 2005: 27). The EU was also involved in the complex structure that was created in 1993–1994 to coordinate aid in Palestine and the 12 working groups, replaced by four Strategy Groups in 2005, which target different aspects of state-building such as economy, governance, infrastructure and social development. As such, the EU and its member states have been directly involved in every facet of the state-building project conducted in Palestine, including the establishment of the PA government’s institutions and structures, such as ministries and public administration.

This involvement unleashed Europeanisation pressures because, through its engagement, the EU has been able to impact the institutional development of Palestine via compliance with institutional directions as well as through change of practices. The legal basis for this has been the Interim Association Agreement signed between the EU and the PLO, on behalf of the PA in 1997. The Action Plan, concluded in 2005 on the basis of this agreement, prioritised structural changes relating to PA institutions, including judicial and electoral reform, effective enforcement of legislation, open, fair and free elections, transparency of public finances and the restructuring of civil service and security (EU-PA Action Plan, 2005). These prescriptions had a strong impact as they were mostly implemented on the ground by the Palestinians, who embarked on the requested domestic reforms, such as the holding of elections in 2006. Another example of EU-induced changes via specific institutional prescriptions (mechanism number 1) has been the pressure exerted on the Palestinian leadership in 2002 for the adoption and entry into force of the Basic Law, legislation on the independence of the judiciary and abolition of the State Security Courts. In 2003, EU pressure also resulted in the revision of the Palestinian Basic Law and the creation of a prime ministerial post. The rationale behind this was to curb the powers concentrated in the hands of Palestinian President Arafat, and this change impacted not only the new institutional design but also the distribution of political power.

Because of contested statehood conditions, and particularly owing to the low degree of internal sovereignty and the lack of well-functioning core state institutions (such as, for example, security institutions), the EU deployed two civilian missions in Palestine in order to help the PA reform its security sector. The first, EUBAM Rafah, was deployed in 2005 at Rafah Crossing Point in order to (a) assist the PA to build capacity on border management and customs, (b) evaluate the PA’s application of the procedures, (c) contribute to confidence building between Israel and the PA, (d) ensure effective border control and, finally, (e) contribute to the liaison between the Palestinian, Israeli and Egyptian authorities in all aspects of border management at Rafah (Council of the European Union, 2005a). The second mission, EUPOL COPPS, provides training, advice and equipment to the Palestinian Civil Police (PCP) and also works closely with Palestinian institutions such as the ministries of justice and interior. Officials of the Civilian Planning and Conduct Capability and the Political and Security Community responsible for EUPOL COPPS underline how they have assisted the drafting of the Code of Conduct on the Use of Force and Firearms (already entered into force) and helped the relevant ministries with drafting the Police Law and the Criminal Procedure Law.^14^ In this regard, the development of EUPOL COPPS has triggered the socialisation of the PCP officers and civil servants into certain practices and styles as well as ‘ways of doing things’ (see mechanism number 3). The missions have also had an impact on the strengthening of internal organisation capacities and staff skills. As an official from the PCP states, ‘We now have the ability to train our people and our own policemen and all this thanks to the EU’.^15^

Yet, contested statehood conditions and the lack of Palestinian control over the territories they claim (linked to Israel’s control of those areas) have had rather negative implications for the operationalisation of both missions on the ground and the impact that the EU has exerted. In the case of EUBAM Rafah, for example, Israel had the upper hand on whether the European monitors would be permitted to reach Rafah and, consequently, whether the border crossing would be open or not. Israel also had the ultimate control of the rest of the crossing points in and out of Gaza. Similarly, EUPOL COPPS also faced limitations because its staff can be present only where the Palestinian police are permitted to operate, and this decision is taken by the Israeli Defense Forces. The deployment of both missions on the ground allowed the EU to have a say and impact on two of the ‘final status’ issues (security and borders) affecting the resolution of the Israeli–Palestinian conflict (Bouris, 2012: 262), but, at the same, the missions’ operationalisation, and the related potential for Europeanisation, has directly been affected because of the realities of the Israeli occupation and Palestine’s lack of internal sovereignty. Because of the restrictions imposed by Israel (see above), a range of training or running of broader projects on border management, human rights, customs and capacity-building could not take place.

Like the TRNC, the Palestinian contested state suffers from a broader underdevelopment, which goes beyond state institutions and touches upon the economy and infrastructure. However, in this case, it is more a result of the low degree of internal sovereignty. Because of the lack of territorial control and developed state institutions, new opportunities for Europeanisation exist, not only in the domains of institutional changes but also in development policies. Palestine is the second biggest recipient of EU development aid after Turkey and has received more than €6 billion since 1994 (European Commission, 2013). Between 1994 and 1998, 40% of European money was channelled into construction, infrastructure and institution building (European Commission, 2013: 88).

At the same time, the low degree of internal sovereignty, manifested in the deficit of Palestinian control over the territories they claim, severely limited the impact of the EU in implementing its development projects in Palestine. The division of the West Bank, in areas A, B and C, has meant that the EU could take initiatives and implement its projects mainly in area A, whereas for projects in areas B and C, prior approval had to be given by Israel. As a result, the EU and the PA were allowed to plan development projects only in 1% of area C, which is a stark example of economic disablement. But even in cases where such an approval was given, Israel would still decide over the future fate of these projects. In March 2012, for example, Štefan Füle presented a list of 82 EU-funded projects worth almost €30 million, which were destroyed by Israel from the burst of the second intifada (2000) until 2011. More recently, and as a response to the EU’s decision to prohibit the awarding of EU grants, loans or prizes to Israeli entities based in the occupied Palestinian territories, the Israeli Minister of Defence instructed Coordination of Government Activities in the Territories to cease joint projects with the EU (Sherwood, 2013). To this end, the realities on the ground make it extremely difficult for both the PA and the EU to reliably plan and implement economic development measures—which also limits the engagement and impact of the EU.

A more political EU-induced redistribution of power has also taken place, which favours elites and actors that are perceived as supportive of a compromised solution to the conflict (mechanism number 2). In 2006, following Hamas’ electoral victory, the EU decided to freeze its direct aid by establishing a Temporary International Mechanism which would channel money directly to people and projects, bypassing the Hamas-led government. This reallocation of resources signalled an EU-informed loss of power for Hamas. Further and since 2007, the EU has engaged only with the Fatah-led West Bank government, which has been perceived as being in favour of negotiations and peace with Israel. The low degree of internal sovereignty had an impact on this EU-induced redistribution of power: the fact that the constituting territorial parts (the West Bank and Gaza) of what is called Palestine are physically disconnected has facilitated the EU’s engagement and empowerment of the Fatah-led West Bank while Gaza has remained under political and also physical isolation.

## Opportunities and limitations for the EU in contested states

Different parameters of contested statehood in northern Cyprus and Palestine have shaped the role and impact of the EU, providing important examples for Europeanisation in contested states. Testing our intervening variable of lack of international recognition in the TRNC, we found that inability of the EU to engage with the authorities of a contested state that suffers limited external sovereignty has not allowed the realisation of the full potential of its aims. At the same time, the international isolation which comes with lack of recognition is also responsible for the low absorption capacity of Turkish Cypriots, which also creates further complications, such as delays in projects and extraordinary needs for supervision. Based on these findings a series of questions emerge for the wider future debate on the EU. For example, what does this compromised engagement mean for the resolution of the related conflict? Recently, in TRNC, the failure of the EU to fulfil its promises has damaged its credibility and ability to mobilise people towards resolution of the dispute, like what happened during the Annan Plan in 2004. Having said that, the ongoing isolation of the TRNC might still be an incentive for locals to support a reunification plan again, as their only way out to full international existence as parts of a reunified Cyprus. Also, does the realisation that political uncertainty compromises EU engagement lead to greater efforts for resolving the conflict? Finally, this article has suggested that the EU’s engagement in contested states is difficult because of the lack of international recognition, but, given the different degrees of external sovereignty that exist across contested states, are there any variations in this difficulty? Are there, for example, contested states where there is more room for engagement and/or influence compared to others, and if yes, why?

We can answer some of these questions by comparing northern Cyprus to Palestine, which differ as far international recognition is concerned. Indeed, a greater external sovereignty in the case of Palestine has meant a much heavier EU involvement. This, however, is not to underestimate the challenges that in this case stem from the low degree of internal sovereignty and ineffective state structures and authority over parts of the self-declared territory, which we tested separately as different intervening variables. Low internal sovereignty has limited the ability of the EU to promote its goals in those areas. The territorial fragmentation of territories controlled by the PA and the simultaneous Israeli control of the most part of self-declared territories of the contested state mean that almost everything has to be approved by Israel first, which has long remained the final arbiter of both the EU initiatives and the Palestinian life. This has been the case with the two EU civilian missions, as well as the EU-funded development and infrastructure projects. Ultimately, this compromises the chances of a successful two-state solution. With many contested states facing challenges in fully controlling their declared territories, our analysis can inform more research on the EU’s ability to engage and how this is limited by the lack of internal sovereignty.

At the same time, however, the unique parameters of contested statehood also offer several opportunities for the role and impact of the EU. In both cases, the EU had the opportunity to address underdevelopment, which comes as a result of contested statehood. In northern Cyprus, the low degree of external sovereignty has resulted in years of international isolation and a considerable gap between what the EU would like to foster and what exists locally, where institutions and policies are acquis incompatible and people remain unfamiliar with EU-promoted policies and practices. This gap has increased Europeanisation pressures upon Turkish Cypriot policies and institutions—through technical and financial assistance—and here, Europeanisation takes place via the change of practices (mechanism number 3) towards development and preparation for EU law implementation. The role of the EU (and therefore the extent of Europeanisation) has not fully materialised because of the challenging diplomatic context of contested statehood, but this limitation does not undermine the EU-induced changes explained so far or the potential for further Europeanisation. Palestine presents a similar story, although it is the limited internal sovereignty that mediates EU engagement and Europeanisation. Here, weak state structures provide room for the Europeanisation of institutions and policies through state-building and, ultimately, the strengthening of internal sovereignty (at least when it comes to institutions). Yet, despite the fact that the international community in 2011 praised the improvement and functioning of Palestinian institutions (see International Monetary Fund, 2011: 66; United Nations (UN), 2011: 1; World Bank, 2010: 5), progress has not been linear^16^ and has faced severe limitations because of Israel’s policies, as well as the inability of the Palestinians to fully control their territories. Despite these limitations, when we look at both cases in comparison, it is clear that the misfit between the domestic status quo and what the EU would like to promote facilitates a certain process of Europeanisation, through changing both ideas and practices (mechanism number 3) as well institutional or policy compliance (in the case of Palestine, mechanism number 1). Assisting the development of contested states might create a short-term intransigence on the ‘other side’ (here, the Greek Cypriots and Israel) but has potential for the long-term chances for peace. In Cyprus, for example, narrowing the gap that exists between the developed south and the relatively underdeveloped north is crucial for a smooth implementation of a federal agreement. Similarly, state-building initiatives in Palestine have been used as a conflict resolution tool, which could eventually help the implementation of a two-state solution.

Beyond development, the lack of internal or external sovereignty offers further opportunities for Europeanisation. In the TRNC, the low degree of external sovereignty has enabled the EU to help individuals and civil society to participate more in international processes. The non-recognition of the TRNC has added to the prioritisation of non-state actors instead of authorities from the contested state, which become diplomatically risky interlocutors. This has meant two things for Europeanisation: first, promotion of unique institutional solutions, whereby public authorities are replaced by technocrats or civil society, and, second, the consequent empowerment of those actors, particularly those that were able to capitalise in their already strong capacity, like the KKTO (mechanism number 2). These are important insights and contribute to the existing literature, which has mostly focused on how the international community engages with civil society in similar conflicts because of its important role in reconciliation (see Berg and Pegg, 2016; Caspersen and Herrberg, 2010). Yet this article looked at civil society beyond reconciliation strictly speaking and added details with regard to the specificities of contested statehood and the role of the EU. These new findings need to be combined with existing knowledge on civil society and reconciliation in order to reflect on how this process of Europeanisation might impact conflict resolution. In Cyprus, civil society continues to promote reconciliation, but this process does not seem to be a result of EU assistance. Indeed, EU officials have explained that they viewed the dominance of the reconciliation agenda in civil society organisations as an unhealthy sign of over-politicisation, which they tried to mend by funding organisations with different aims.^17^ Consequently, the relation between civil society promotion and reconciliation in contested states in particular should be problematised more systematically. At the same time, EU processes and actors also need to be examined in more detail. While this study has offered evidence that there exist opportunities for the international integration of locals from contested states, whether certain European processes or institutions are more open to contested states is a question that needs further investigation.

What is also noteworthy is that the case of Palestine seems to be the opposite of the TRNC as far as state institutions are concerned: while the low degree of external sovereignty results in EU avoidance of state institutions in northern Cyprus, the low degree of internal sovereignty leads to engagement with and assistance of state institutions in Palestine. This is also because Palestine enjoys greater external sovereignty in comparison with the TRNC. The main objective is to help Palestine address the absence of an effective state apparatus and to build a state which would eventually contribute to the resolution of the conflict. EU assistance might have allowed Palestine to move up the sovereignty ‘ladder’ as far as effective governance is concerned; however, ongoing problems of territorial control mean that Palestine continues to display low internal sovereignty. This outcome seems to suggest that while governance incapacity offers opportunities for Europeanisation, the other aspect of limited internal sovereignty, that is, ineffective territorial control, limits EU engagement.

## Conclusion

Preoccupied with conventional states, the European studies literature has neglected contested states. This article has addressed this gap in the literature through a comparative discussion that analysed how the lack of international recognition, territorial control and effective government in contested states mediate the engagement and impact of the EU. Our findings—a major empirical contribution in their own—make clear that parameters of contested statehood compromise the involvement of the EU because of its inability to deal with contested state authorities and/or engage in territories that are under the control of the parent/reference state rather than the contested state. At the same time, however, there exist unique opportunities for the EU to make an impact, especially through the assistance of state-building and broader development, as well as international integration. These findings and the proposed conceptual framework from which they emanate are important for understanding the ability of the EU to exert an influence beyond traditional intergovernmental contexts and promote security and reconciliation. Yet, with conflict resolution being the ultimate aim of the EU and other international actors in most contested states, this study aspires to encourage more research on the links between international engagement, contested statehood and the promotion of security and reconciliation.

## Notes

1. Parent or reference state suggests a recognised state that is part of a dispute with a self-declared, contested state (such as the Republic of Cyprus vs Turkish Republic of Northern Cyprus (TRNC) or Israel vs Palestine).

2. For a more in-depth discussion on ‘earned sovereignty’, see Kolstø and Blakkisrud (2012), Scharf (2004) and Williams and Pecci (2004).

3. Interview with European Commission Official ‘C’, Brussels, May 2009.

4. Interview with Turkish Cypriot Chamber of Commerce (KTTO) Member, Brussels, 2009.

5. Interview with European Commission Official A, Brussels, May 2009.

6. Interview with Member of the European Parliament, Brussels, May 2009.

7. Interview with Turkish Cypriot Official A, 2009

8. Email interview with Turkish Cypriot Official B, 2014.

9. Interview with European Commission Official D, Brussels, May 2009.

10. Interview with European Commission Official A, Brussels, May 2009.

11. Email Interview with Turkish Cypriot Official B, 2014

12. Interview with European Commission Official B, Brussels, May 2009.

13. Interview with KTTO Member, Brussels, May 2009; interview with European Union (EU) Official E, Brussels, May 2009; interview with Turkish Cypriot official, Nicosia, September 2009.

14. Interview with a EUPOL COPPS official, Ramallah, 19 June 2015.

15. Interview with a Palestinian Civil Police official, Ramallah, 29 May 2010.

16. It could be argued that there are three periods of state-building in Palestine: the first is between 1993 and 2000 which was interrupted by the burst of the second intifada; the second period of state-building started in 2002/2003 with the Roadmap until the 2006 elections; the third one, from 2007 until today, where because of the division of West Bank Fatah-led government and the Hamas-led Gaza Strip, the state-building project has mainly focused on the West Bank.

17. Interview with European Commission Official D, Nicosia, September 2009.
